# Ethnopharmacological Uses, Phytochemistry, and Pharmacological Properties of* Croton macrostachyus* Hochst. Ex Delile: A Comprehensive Review

**DOI:** 10.1155/2017/1694671

**Published:** 2017-10-12

**Authors:** Alfred Maroyi

**Affiliations:** Medicinal Plants and Economic Development (MPED) Research Center, Department of Botany, University of Fort Hare, Private Bag X1314, Alice 5700, South Africa

## Abstract

*Croton macrostachyus* is widely used as herbal medicine by the indigenous people of tropical Africa. The potential of* C. macrostachyus* as herbal medicine, the phytochemistry, and pharmacological properties of its parts used as herbal medicines are reviewed. The extensive literature survey revealed that* C. macrostachyus* is traditionally used to treat or manage at least 81 human and animal diseases and ailments. The species is used as herbal medicine for diseases and ailments such as abdominal pains, cancer, gastrointestinal disorders, malaria, pneumonia, sexually transmitted infections, skin infections, typhoid, and wounds and as ethnoveterinary medicine. Multiple classes of phytochemicals such as alkaloids, amino acids, anthraquinones, carbohydrates, cardiac glycosides, coumarins, essential oil, fatty acids, flavonoids, phenolic compounds, phlobatannins, polyphenols, phytosteroides, saponins, sterols, tannins, terpenoids, unsaturated sterol, vitamin C, and withanoides have been isolated from the species. Pharmacological studies on* C. macrostachyus* indicate that it has a wide range of pharmacological activities such as anthelmintic, antibacterial, antimycobacterial, antidiarrhoeal, antifungal, anticonvulsant and sedative, antidiabetic, anti-inflammatory, antileishmanial, antioxidant, antiplasmodial, and larvicidal effects.* Croton macrostachyus* has potential as a possible source of a wide range of pharmaceutical products for the treatment of a wide range of both human and animal diseases and ailments.

## 1. Introduction

*Croton macrostachyus* Hochst. ex Delile is a species of the genus* Croton* L., Euphorbiaceae family, commonly known as the spurge family.* Croton macrostachyus* is a medium sized, drought-deciduous pioneer tree which regenerates naturally in less productive sites including forest edges, mountain slopes, and waste grounds under a wide range of ecological conditions [1–3].* Croton macrostachyus* is regarded as a multipurpose tree by subsistence farmers in Ethiopia, Kenya, and Tanzania [3–6], as it is often grown and managed in home gardens for provision of several ecosystem goods and services. In Ethiopia, for example,* C. macrostachyus* is a major tree intercropped in agroecosystems in order to increase soil productivity in midaltitude and semiarid areas [7]. There is also tremendous interest in the medicinal uses and pharmacological properties of* C. macrostachyus* throughout its distributional range in tropical Africa [8–11]. Research by these authors revealed that* C. macrostachyus* is an important medicinal plant in tropical Africa with potential of providing important pharmaceutical products to be used by rural and urban communities who rely on herbal medicines for primary healthcare. Integration of traditional medicine and modern medicine has been recommended by the World Health Organization (WHO) since 1978 [12], mainly because traditional medicines are perceived to be more affordable, accessible, and acceptable to poor rural and urban communities and those living in marginalized areas [13]. Considering the documented ethnomedicinal uses of* C. macrostachyus* in tropical Africa [8–11], certainly the species has potential in playing an important role in the primary healthcare of communities throughout its distributional range. It is therefore important to assess if there is correlation between the ethnomedicinal uses of* C. macrostachyus* and the recent documented phytochemical and pharmacological properties of the species. Therefore, the present review collates the fragmented information on traditional uses, phytochemistry, pharmacology, and toxicology of the species. It is hoped that this information will highlight the importance of* C. macrostachyus* as a potential source of a wide range of pharmaceutical products in tropical Africa and will provide a new direction for researchers in the future.

## 2. Methodology of the Review

*Croton macrostachyus* and other historical names and synonyms of the species were used as the keywords in searching the major databases including Web of Science, Scopus, Google Scholar, Science Direct, BioMed Central (BMC), PubMed, and Springerlink documenting traditional uses, medicinal uses, ethnobotany, ethnomedicinal uses, ethnopharmacology, pharmacology, phytochemistry, and therapeutic value of the species. Additional literature, including preelectronic literature such as dissertations, theses, and other grey materials were sourced from the University of Fort Hare library in South Africa.

## 3. Botanical Profile, Taxonomy, and Distribution of* Croton macrostachyus*

**Figure 1:**
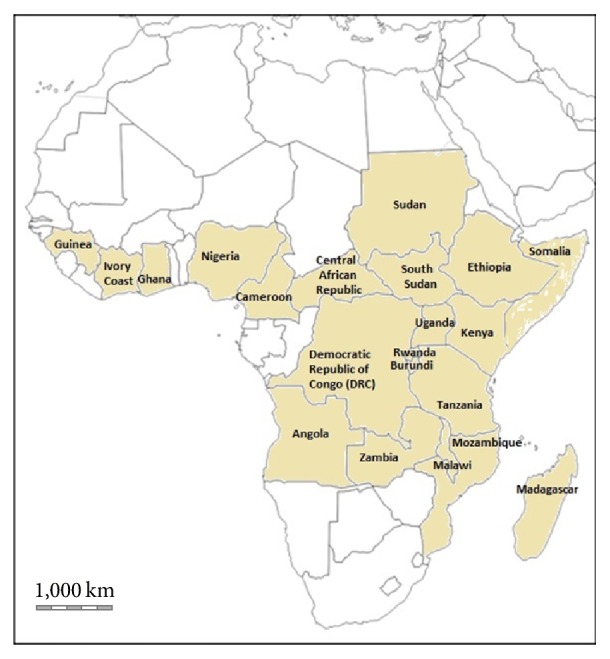
Distribution of* Croton macrostachyus* in tropical Africa.

The genus name “*Croton*” was derived from a Greek word “kroton,” a tick, referring to thick smooth seeds, a common feature of most* Croton* species which belong to the Crotonoideae subfamily of the Euphorbiaceae family [14]. The specific name “*macrostachyus*” is a contraction of two words, the Greek word “macro” meaning large and “stachyus” relating to the spike, hence, a species characterized by large spikes [15]. Historical names or synonyms of* C. macrostachyus* are* C. acuminatus* R. Br.,* C. butaguensis* De Wild.,* C. guerzesiensis* Beille ex A. Chev.,* C. macrostachyus* var.* mollissimus* Chiov.,* Oxydectes macrostachya* (Hochst. ex Delile) Kuntze, and* Rottlera schimperi* Hochst. & Steud. (http://www.theplantlist.org/tpl1.1/record/kew-50457).* Croton macrostachyus* is commonly known as “broad-leaved croton” or “rush foil” in English, “bisana” in Amharic in Ethiopia, and “msinduzi” in Swahili in east Africa [11, 16–24].* Croton macrostachyus* is widely distributed in tropical Africa, from Guinea east to Ethiopia and Somalia, south to Angola, Mozambique, and Madagascar (Figure 1). The species has been reported to occur in Angola, Burundi, Cameroon, Central African Republic, Democratic Republic of Congo (DRC), Ethiopia, Ghana, Guinea, Ivory Coast, Kenya, Madagascar, Malawi, Mozambique, Nigeria, Rwanda, Somalia, South Sudan, Sudan, Tanzania, Uganda, and Zambia [3, 25]. According to Mairura [11],* C. macrostachyus* is common in secondary forests, especially on forest edges and along rivers or lakes, in moist or dry evergreen upland forest, woodland, wooded grassland, bushland, and along roadsides, often on soils of volcanic origin at altitude between 200 to 3400 m above sea level and mean annual rainfall between 150 mm and 1200 mm.

*Croton macrostachyus* is a monoecious or dioecious, deciduous, medium sized tree up to 30 m tall [2]. The bole is cylindrical up to 100 cm in diameter with grey to grey-brown bark, finely fissured, and cracked, inner bark pale brown to reddish brown with a peppery smell [1]. The leaves are alternate, simple, turning orange before falling with linear stipules, up to 15 mm long [1]. The petiole is up to 20 cm long with two stalked glands at top [2]. The leaf blade is ovate-elliptical to almost circular, up to 25 cm × 20 cm in size with a cordate base and acuminate apex acuminate with irregularly toothed margins, densely stellate hairy on both sides, and whitish green beneath [1]. The inflorescence is a slender, terminal raceme up to 35 cm long, either with only male or female flowers or male and female flowers variably mixed [2]. Male flower has a pedicel which is 3–10 mm long with campanulate calyx with ovate to triangular lobes, 2.5–3.5 mm long with densely white hairy margins [1]. The petals are oblong to oblanceolate, 3–4.5 mm long and have 15–17 free stamens [1]. The female flower has a pedicel which is 2–4 mm long, fleshy, calyx as in male flowers but lobes more triangular, persistent in fruit, petals linear, or absent up to 1.5 mm long [1]. The ovary is superior, rounded, densely stellate hairy with three styles, 3–6 mm long, twisted, and curved [2]. The fruit is a globular capsule, 8–12 mm in diameter with a centrally depressed apex, whitish to pale greyish brown in colour. The seeds are ellipsoid, 6–8 mm × 4–5.5 mm in size, flattened, and cream-coloured [1].

## 4. Traditional and Contemporary Uses of* Croton macrostachyus*

The bark, fruits, leaves, roots, and seeds of* C. macrostachyus* are reported to possess diverse medicinal properties and cure various human and animal diseases and ailments throughout the distributional range of the species (Table 1).* Croton macrostachyus* is used as herbal medicine for at least 61 and 20 human and animal diseases and ailments, respectively (Table 1). There is cross-cultural agreement among ethnomedicinal uses of* C. macrostachyus* throughout its distributional range, and there is also a high degree of medicinal use consensus (recorded in at least two countries) for bleeding, blood clotting, cancer, constipation, diarrhoea, epilepsy, malaria, pneumonia, purgative, ringworm, skin diseases or infections, stomach ache, typhoid, worm expulsion, and wounds (see Table 1).

In Cameroon, Ethiopia, Kenya, Rwanda, Somalia, Tanzania, and Uganda, leaf decoction, infusion or maceration, stem bark, or root bark of* C. macrostachyus* is taken as a purgative and vermifuge (Table 1).* Croton macrostachyus* is also used in combination with other plant species. For example, in Ethiopia, a bark decoction of* C. macrostachyus* is often mixed with roots of* Cucumis ficifolius* A. Rich. as remedy for abdominal pain [28]. Leaf decoction of* C. macrostachyus* is often mixed with leaves of* Trichilia* spp. and* Rhamnus prinoides* L'Hérit. as remedy for diarrhoea and dysentery in humans and to repel external parasites in livestock [48]. Research by Mesfin et al. [60] and Bekele and Reddy [61] revealed that crushed leaves of* C. macrostachyus* are boiled in water mixed with* Allium sativum* L. bulb roasted with butter; the concoction is allowed to brew overnight and taken orally the following day as remedy for malaria. For skin diseases and wounds, the fruit decoction of* C. macrostachyus* is mixed with leaves of* Hagenia abyssinica* (Bruce) J.F.Gmel. and applied topically on affected body parts [69] while leaf sap of* C. macrostachyus* is mixed with coconut (*Cocos nucifera* L.) milk and applied on wounds [44] or a leaf decoction mixture of* C. macrostachyus*,* Cynoglossum lanceolatum* Forssk., and* Dodonaea viscosa* subsp.* angustifolia* (L.f.) J.G.West is applied on wounds [17]. According to Teklehaymanot et al. [17], bark decoction of* C. macrostachyus* is mixed with stems of* Glinus lotoides* L. as herbal medicine for tapeworms while bark decoction of* C. macrostachyus* is mixed with leaves of* Juniperus procera* Hochst. ex Endl., stems of* Eragrostis tef* (Zucc.) Trotter, and roots of* Cyphostemma cyphopetalum* (Fresen.) Desc. ex Wild & Drummond,* Solanum anguivi* Lam., and* Solanum marginatum* L. f. as herbal medicine for rabies in Ethiopia. Research by Nahayo et al. [72] revealed that leaf decoction of* C. macrostachyus* mixed with leaves of* Baccharoides calvoana* subsp.* meridonalis* (Wild) Isawumi, El-Ghazaly & B.Nord,* Vernonia auriculifera* Hiern, and* Salvia nilotica* Juss. ex Jacq. or bark decoction of* C. macrostachyus* mixed with bark of* Morella salicifolia* subsp.* mildbraedii* (Engl.) Verdc. & Polhill,* Olea capensis* subsp.* macrocarpa* (C.H.Wright) Verdc., and* Elaeis guineensis* Jacq. is used for worm expulsion in Rwanda. The leaves of* C. macrostachyus* are used by farmers in Kenya as biological pest control when mixed with tobacco (*Nicotiana tobacuum* L.) and boiled overnight [57]. The resultant mixture is used as a biological pesticide for the control of maize stalk borers and aphids [57].

Table 1 provides a summary of ethnomedicinal uses and plant parts of* C. macrostachyus* used among diverse ethnic groups in tropical Africa. In Cameroon, root decoction of* C. macrostachyus* is used as purgative [65], while bark and leaf decoctions are used as remedies for epilepsy, insomnia, and typhoid [50, 63, 74]. In Ethiopia,* C. macrostachyus* has many uses including abdominal pain, abortifacient, amoebiasis, antidote for scorpion, and snake venom, anthrax, ascariasis, cancer, constipation, diarrhoea, dysentery, epilepsy, jaundice, leprosy, malaria, ringworm, sexually transmitted infections (STIs), skin diseases, stomach ache, tapeworms, typhoid, and wounds [17, 20, 26, 28–32, 34, 35, 38, 40, 44, 45, 49–52, 55–57, 60, 61, 63, 65, 67, 69, 72, 74, 82]. In Kenya,* C. macrostachyus* bark juice, leaf, and root decoction is used as remedy for backache, bleeding, cancer, colds, cough, diarrhoea, dysmenorrhoea, east coast fever, malaria, measles, obesity, pneumonia, ringworm, skin diseases, typhoid, warts, and wounds [37, 39, 46, 58, 59, 62, 68]. Research by Mazzanti et al. [66] revealed that seeds are used as purgatives in Somalia. In Tanzania, fruit and decoction of* C. macrostachyus* are used as purgative [16], while fruit, leaf, and root decoctions are used as remedies for abdominal pain, constipation, diabetes, ringworm, skin infections, sores, and worm expulsion [16, 27, 43]. In Uganda,* C. macrostachyus* bark and root decoctions are used as remedies for headache, stomach ache, and worms [54, 71]. Bark, leaf, root, and twig decoctions of* C. macrostachyus* are used as ethnoveterinary medicine in Ethiopia and Kenya for abdominal pain, blackleg, bleeding, bloat, colic, constipation, dermatophilosis, epilepsy, fever, rabies, rectum prolapsed, ringworm, scabies, skin diseases, warts, and wounds [17, 21, 23, 35, 36, 38, 44, 48, 68, 75–78].

In Kenya, Tanzania, and Uganda,* C. macrostachyus* is commonly planted as an ornamental or shade tree in villages and the tree is also used as a shade-bearer on coffee plantations and other crops [16, 57, 80]. The wood is used in Cameroon, Ethiopia, Kenya, Tanzania, and Uganda to make tool handles, small stools, boxes, crates, and plywood, as flooring and building material and in carpentry [16, 40, 57, 63, 65, 80]. The wood is used as fuel that burns even when green but produces a rather unpleasant spicy odour and much smoke; it is also used to make charcoal [11, 16, 63, 65, 80]. Due to its drought hardiness and fast growth,* Croton macrostachyus* is considered useful for afforestation of shifting sand dunes, degraded waste land, hill slopes, ravines, and lateritic soils [11].

## 5. Phytochemistry

**Figure 2:**
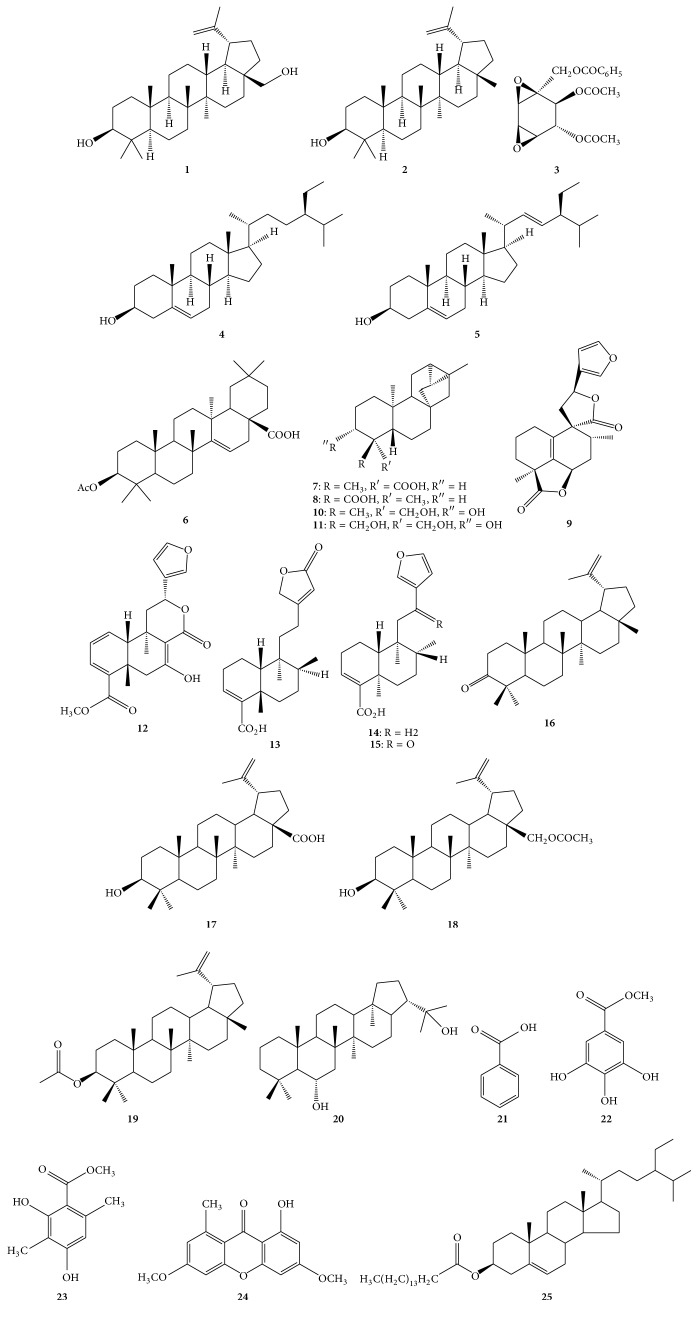
Chemical structures of major compounds isolated from leaves, roots, and stems of* Croton macrostachyus*.

Multiple classes of phytochemicals including alkaloids, amino acids, anthraquinones, carbohydrates, cardiac glycosides, coumarins, essential oil, fatty acids, flavonoids, phenolic compounds, phlobatannins, polyphenols, phytosteroides, saponins, sterols, tannins, terpenoids, unsaturated sterol, vitamin C, and withanoides have been identified from* C. macrostachyus* fruits, leaves, stem bark, and twigs [24, 30, 47, 55, 83–89]. Addae-Mensah et al. [83] isolated betulin** 1**, lupeol** 2**, crotepoxide** 3**, *β*-sitosterol** 4**, and stigmasterol** 5** from stem barks and twigs of* C. macrostachyus* (see Table 2, Figure 2). Kapingu et al. [90] isolated 3*β*-acetoxy taraxer-14-en-28-oic acid** 6**, trachyloban-19-oic acid** 7**, trachyloban-18-oic acid** 8**, neoclerodan-5,10-en-19,6*β*; 20,12-diolide** 9**, 3*α*,19-dihydroxytrachylobane** 10**, and 3*α*,18,19-trihydroxytrachylobane** 11** from roots of* C. macrostachyus*. Tane et al. [91] isolated crotepoxide** 3** and crotomacrine** 12** from fruits of* C. macrostachyus*. Tene et al. [92] isolated betulin** 1**, lupeol** 2**, floridolide A** 13**, hardwickic acid** 14**, and 12-oxo-hardwickic acid** 15** from stem bark of* C. macrostachyus*. Tala et al. [93] isolated and identified betulin** 1**, lupeol** 2**, *β*-sitosterol** 4**, stigmasterol** 5**, lupenone** 16**, betulinic acid** 17**, 28-O-acetylbetulin** 18**, lupeol acetate** 19**, zeorin** 20**, benzoic acid** 21**, methyl gallate** 22**, methyl 2,4-dihydroxy-3,6-dimethylbenzoate** 23**, lichexanthone** 24**, and *β*-sitosterol palmitate** 25** from twigs of* C. macrostachyus*.

## 6. Pharmacological Activities

A number of pharmacological activities of* C. macrostachyus* have been reported in literature justifying some of its ethnomedicinal uses listed in Table 1. These pharmacological activities include anthelmintic [30, 95], antibacterial [53, 55, 62, 85, 92, 96–100], anticonvulsant and sedative [50], antidiabetic [88], antidiarrhoeal [24], antifungal [62, 86, 92, 101], anti-inflammatory [84, 102], antileishmanial [94], antioxidant [86], antiplasmodial [87, 89, 103, 104], antimycobacterial [105], larvicidal [19, 50], and cytotoxicity [27, 43, 73, 96, 104–108].

### 6.1. Anthelmintic

Eguale et al. [30] evaluated anthelmintic activities of crude aqueous and hydroalcoholic extracts of the seeds of* C. macrostachyus* on eggs and adult tapeworms* (Haemonchus contortus)*. Both aqueous and hydroalcoholic extracts of* C. macrostachyus* induced statistically significant egg hatching inhibition (*p* < 0.05) with aqueous extract requiring maximum concentration of 0.5 mg/ml to induce 100% egg hatch inhibition while the hydroalcoholic extracts did not induce complete inhibition at highest concentration tested of 2 mg/ml. The aqueous extract of* C. macrostachyus* induced 50% inhibition (ED_50_) at 0.10 mg/ml which was at a lower concentration than the hydroalcoholic extract at ED_50_ value of 0.32 mg/ml [30]. After 24 hours of exposure of adult* Haemonchus contortus* to different concentration of plant extracts, hydroalcoholic extracts of the species produced mortality of adult* Haemonchus contortus* to the level of 90% at concentration of 8 mg/ml while aqueous extract produced only 36.67% at the same concentration [30]. Similarly, Aleme et al. [95] evaluated the anthelmintic effects of crude aqueous extracts of the leaves of* C. macrostachyus* against adults of live* Haemonchus contortus* and the efficacy of these crude aqueous extracts was determined based on the mortality rate of the adult parasite. The efficacy at 4 mg/ml of the aqueous extracts of* C. macrostachyus* was 70% against the adult stage of* Haemonchus contortus* and the efficacy of the positive control, albendazole, against the adult parasite was dose-dependent and all the adult worms were dead at a concentration of 0.5 mg/ml within 24 hours [95]. These findings indicate that* C. macrostachyus* has potential anthelmintic effect and could be used as an inexpensive and eco-friendly alternative to controlling tapeworm infections.

### 6.2. Antibacterial

Geyid et al. [55] evaluated antibacterial activities of methanol, petroleum ether, and aqueous fruit extracts of* C. macrostachyus* against* Bacillus cereus*,* Escherichia coli*,* Neisseria gonorrhoea*,* Salmonella typhi*,* Salmonella typhimurium*,* Shigella dysentery*,* Shigella flexineri*,* Staphylococcus aureus*,* Streptococcus pneumoniae*, and* Streptococcus pyogenes* using the agar dilution method. Methanol fruit extract of C. macrostachyus inhibited growth of* Neisseria gonorrhoeae* at four levels of concentration, that is, 250, 500, 1000, and 2000 *μ*g/ml. Mesfin et al. [85] evaluated antibacterial activities of chloroform, n-butanol, and aqueous fractions of* C. macrostachyus* against* Neisseria gonorrhoeae* using the agar dilution method. Chloroform and n-butanol fractions of* C. macrostachyus* were more active with minimum inhibitory concentration (MIC) values ranging from 125 to 250 *μ*g/mL [85]. Wagate et al. [96] also evaluated antibacterial activities of leaf and root methanolic extracts of* C. macrostachyus* against* Bacillus cereus*,* Escherichia coli*,* Pseudomonas aeruginosa*, and* Staphylococcus aureus* using the broth dilution method with benzylpenicillin and streptomycin as positive controls. The methanolic extracts of* C. macrostachyus* were the most active against* Bacillus cereus* with minimum inhibitory concentration (MIC) value of 15.6 mg/mL and MIC value of 250 mg/mL against both* Escherichia coli* and* Pseudomonas aeruginosa* [96]. In another study, Wagate et al. [97] evaluated antibacterial activities of methanol leaf and root extracts of* C. macrostachyus* against* Bacillus cereus*,* Escherichia coli*,* Micrococcus lutea*, and* Pseudomonas aeruginosa* using broth dilution method with benzylpenicillin and streptomycin as positive controls.* Croton macrostachyus* methanol leaf and root extracts were active against* Bacillus cereus* with minimum inhibitory concentration (MIC) value of 15.6 mg/mL as well as* Escherichia coli* and* Pseudomonas aeruginosa*, both with MIC value of 250 mg/mL [97]. Tene et al. [92] also evaluated antibacterial activities of the ethanol stem bark extract of* C. macrostachyus* and five compounds isolated from the stem bark of the species, namely, betulin** 1**, lupeol** 2**, floridolide A** 13**, hardwickic acid** 14**, and 12-oxo-hardwickic acid** 15** against* Klebsiella pneumoniae*,* Salmonella typhi*, and* Staphylococcus aureus*. The ethanol stem bark extract demonstrated some activity with the minimum bactericidal concentration (MBC) values ranging from 31.25 to 500 *μ*g/ml; the best activity of 31.25 *μ*g/ml was against* Staphylococcus aureus*. The compounds demonstrated some activity with the minimum bactericidal concentration (MBC) values ranging from 15.62 to >1000 *μ*g/ml. The best activity was demonstrated by betulin** 1** with MIC value of 15.25 *μ*g/ml against* Salmonella typhi* and* Staphylococcus aureus*, and MIC value of 31.25 *μ*g/ml against* Klebsiella pneumoniae*, while hardwickic acid** 14** had MIC value of 31.25 *μ*g/ml against* Staphylococcus aureus* and 12-oxo-hardwickic acid** 15** had MIC value of 62.5 *μ*g/ml against* Staphylococcus aureus* [92].

Belay et al. [98] evaluated antibacterial activities of volatile fractions of* C. macrostachyus* fruits against* Bacillus cereus*,* Citrobacter* spp.,* Escherichia coli*,* Klebsiella pneumonia*,* Listeria monocytogenes*,* Proteus mirabilis*,* Pseudomonas aeruginosa*,* Salmonella paratyphi*,* Shigella dysenteriae*,* Staphylococcus aureus*, and* Streptococcus pyogenes* using dimethyl sulfoxide (DMSO) and Tween-80 as negative controls and gentamicin as positive control. Antibacterial activity was demonstrated with minimum inhibitory concentration (MIC) values ranging from 0.1 to 12.5 *μ*g/ml and minimum bactericidal concentration (MBC) values ranging from 0.2 to 25 *μ*g/ml [98]. Taye et al. [99] also evaluated antibacterial activities of aqueous and methanol leaf extracts of* C. macrostachyus* against* Escherichia coli*,* Proteus vulgaris*,* Pseudomonas aeuruginosa*,* Staphylococcus aureus*, and* Streptococcus pyogenes* using the agar well diffusion method with ciprofloxacin and amoxicillin as positive controls. Antibacterial activity was demonstrated by methanol leaf extract against* Streptococcus pyogenes* with minimum bacterial concentration (MBC) value of 7.81 mg/mL. Mesfin et al. [53] also evaluated antibacterial activities of chloroform, n-butanol, and aqueous leaf extracts of* C. macrostachyus* against* Neisseria gonorrhoeae* using the agar dilution method. Chloroform and n-butanol fractions were identified to be more active with minimum inhibitory concentration (MIC) values between 125 and 250 *μ*g/ml [53]. Similarly, Sendeku et al. [100] evaluated antibacterial activities of chloroform, ethanol and methanol leaf extracts of* C. macrostachyus* using the agar well diffusion and broth dilution assay methods against* Escherichia coli*,* Klebsiella pneumonia*,* Salmonella pneumonia*,* Shigella flexneri*, and* Staphylococcus aureus*. The leaf extract showed some activity with minimum inhibitory concentrations (MIC) varying from 3.75 to 30.0 mg/ml and minimum bactericidal concentrations (MBC) varying from 7.5 to 40.0 mg/ml [100]. Recently, Obey et al. [62] evaluated antibacterial activities of methanol, ethyl acetate, and butanol stem bark extracts and purified lupeol** 2** isolated from* C. macrostachyus* against* Escherichia coli*,* Salmonella typhi*,* Klebsiella pneumoniae*,* Enterobacter aerogenes*, and* Listeria monocytogenes* using the agar well diffusion method. The most promising broad scale antibacterial activity against all the studied pathogens was shown by the ethyl acetate extract with minimum inhibitory concentrations (MICs) ranging from 125 to 250 mg/mL and lupeol 2 had the lowest MIC value of 125 mg/mL against* Klebsiella pneumoniae*. These antibacterial activities displayed by different extracts [53, 55, 62, 85, 92, 96–100] somehow confirm the potential of* C. macrostachyus* in the treatment and management of bacterial infections as detailed in Table 1.

### 6.3. Antimycobacterial

Gemechu et al. [105] evaluated antimycobacterial activities of methanolic leaf extracts of* C. macrostachyus* against* Mycobacterium tuberculosis* and* Mycobacterium bovis* strains using 96 wells of microplate with the help of visual Resazurin Microtiter assay. The methanolic leaf extracts of* C. macrostachyus* demonstrated antimycobacterial activity with minimum inhibitory concentration (MIC) values ranging from 12.5 to 100 *μ*g/mL. The results of this study demonstrate that* C. macrostachyus* has potential as herbal medicine in the treatment and management of tuberculosis, a leading cause of death in sub-Saharan Africa [109]. However, further investigations are needed aimed at identifying chemical constituents of* C. macrostachyus* responsible for these activities and their mode of action.

### 6.4. Antifungal

Tene et al. [92] evaluated antifungal activities of the ethanol stem bark extract of* C. macrostachys* and chemical compounds isolated from the stem bark of the species, namely, betulin** 1**, lupeol** 2**, floridolide A** 13**, hardwickic acid** 14**, and 12-oxo-hardwickic acid** 15** against* Candida albicans*,* Candida krusei*, and* Cryptococcus neoformans*. The ethanol stem bark extract demonstrated some activity with the minimum fungicidal concentration (MFC) values ranging from 62.5 to 1000 *μ*g/ml, and the best activity of 62.5 *μ*g/ml was against* Candida albicans*. The compounds demonstrated some activity with the minimum fungicidal concentration (MFC) values ranging from 7.81 to >1000 *μ*g/ml. The best activity was demonstrated by 12-oxo-hardwickic acid** 15** with MIC value of 7.81 *μ*g/ml against* Candida albicans*, MIC value of 31.25 *μ*g/ml against* Candida krusei* and* Cryptococcus neoformans*, and hardwickic acid** 14** had MIC value of 62.5 *μ*g/ml against* Candida albicans* [92]. Abera et al. [101] also evaluated antifungal activity of aqueous and ethanol extracts of* C. macrostachyus* against* Colletotrichum kahawae*, a fungus that causes coffee berry disease.* Croton macrostachyus* aqueous and ethanol extracts reduced radial growth of* Colletotrichum kahawae* in ethanol and aqueous extracts by 68% and 88%, respectively. This study indicated the possible use of* C. macrostachyus* extracts as an alternative means of coffee berry disease management [101]. Teugwa et al. [86] evaluated antifungal activities of aqueous and methanol leaf extracts of* C. macrostachyus* against* Trichophyton rubrum*,* Trichophyton soudanense*, and* Trichophyton violaceum* using the agar dilution method with amphotericin as positive control. All extracts tested showed antifungal activity against the three* Trichophyton* species tested, with minimum inhibitory concentration (MIC) and minimum fungicidal concentrations (MFC) varying from 17.50 to 27.50 and 20 to 30 mg/ml, respectively. Recently, Obey et al. [62] evaluated antifungal activities of methanol, ethyl acetate, and butanol stem bark extracts and purified lupeol 2 isolated from* C. macrostachyus* against* Candida albicans* using the agar well diffusion method. The most promising broad scale antifungal activity was shown by the ethyl acetate extract with minimum inhibitory concentrations (MICs) of 500 mg/mL and lupeol** 2** had MIC value of 500 mg/mL [62].

### 6.5. Antidiarrhoeal

Degu et al. [24] evaluated the antidiarrheal activities of chloroform and methanol leaf extracts of* C. macrostachyus* using the castor oil induced diarrheal model, charcoal meal test and antienteropooling test in mice. The test groups received various doses (300, 400, and 500 mg/kg and an additional dose of 1000 mg/kg for the aqueous fraction) of the fractions, whereas positive controls received either loperamide (3 mg/kg) or atropine (5 mg/kg) and negative controls received vehicle (10 ml/kg). In the castor oil induced model, the chloroform (at all test doses) and methanol (at 400 and 500 mg/kg) fractions delayed diarrheal onset and decreased stool frequency and weight of faeces. The chloroform and methanol fractions produced dose-dependent decline in the weight and volume of intestinal contents while the aqueous fraction did not have a significant effect [24]. These authors also found that all the fractions produced antimotility effect either at all doses (chloroform fraction) or at middle and higher doses (methanol and aqueous fractions).

### 6.6. Anticonvulsant and Sedative

Bum et al. [50] evaluated anticonvulsant effects of crude extracts of* C. macrostachyus* using mice model (maximal electroshock (MES), strychnine (STR), pentylenetetrazol (PTZ), picrotoxin (PIC), isonicotinic hydrazide acid (INH))-induced convulsions and diazepam-induced sleep in assessing the sedative effects.* Croton macrostachyus* at the doses of 34 and 67 mg/kg protected 80, 80, 80, and 60% of mice from PIC, STR, PTZ, and MES-induced seizures, respectively [50].* Croton macrostachyus* also delayed the onset to seizures in the INH test. The decoctions of* C. macrostachyus* possess sedative and anticonvulsant activities and these results corroborate the use of the species as herbal medicine for epilepsy in Ethiopia [36] and epilepsy and insomnia in Cameroon [50].

### 6.7. Antidiabetic

Arika et al. [88] evaluated* in vivo* hypoglycemic activity of aqueous leaf extracts of* C. macrostachyus* in male Swiss white albino mice. Aqueous leaf extract of* C. macrostachyus* was intraperitoneally and orally administered to alloxan (180.9 mg/kg; intraperitoneally) induced diabetic mice at different doses of 25 mg/kg body weight (bwt), 48.4 mg/kg bwt, 93.5 mg/kg bwt, 180.9 mg/kg bwt, and 350 mg/kg bwt and the effects on blood glucose levels investigated. Treatment of diabetic mice with doses of the leaf extract resulted in significantly lower levels of fasting blood glucose and the effects of the leaf extract were comparable with the conventional drugs [88]. Therefore, the results suggest that* C. macrostachyus* leaf extract is a potent hypoglycemic agent and this validates the use of root decoction as herbal medicine for diabetes in Tanzania [43].

### 6.8. Anti-Inflammatory

Kamanyi et al. [84] evaluated the antinociceptive and anti-inflammatory activities of the aqueous and methylene chloride/methanol stem bark extracts of* C. macrostachyus*. The extracts were administered orally at the doses of 150, 300, and 600 mg/kg and examined against pain induced by acetic acid, formalin, and pressure and against inflammation induced by carrageenan, histamine, and formalin. Results obtained by Kamanyi et al. [84] showed that both extracts induced dose-dependent reduction in the number of abdominal constrictions induced by acetic acid, and the three doses of the two extracts also reduced the two phases of pain induced by formalin. At the dose of 600 mg/kg, the aqueous and the methylene chloride/methanol extracts exhibited analgesic activity against pressure-induced pain. The two extracts also exhibited anti-inflammatory activity, the methylene chloride/methanol extract being the most active inhibited acute inflammation induced by carrageenan, histamine, and formalin, and both extracts reduced the chronic inflammation induced by formalin [84]. Nguelefack et al. [102] also evaluated the antinociceptive properties of the methanol/methylene chloride extracts of the stem bark of* C. macrostachyus* using mice models of persistent inflammatory and neuropathic pain and also assessed its mechanism of action. The methanol/methylene chloride extract was tested on Complete Freund Adjuvant- (CFA-) induced persistent thermal and mechanical pain, neuropathic pain induced by partial sciatic nerve ligation (PSNL), prostaglandin E_2_- (PGE_2_-) induced acute mechanical hyperalgesia, as well as on nociception induced by capsaicin in mice. Mechanical hyperalgesia was assessed using von Frey hair in awake mice. The mechanism of action of methanol/methylene chloride extract was evaluated by using glibenclamide on PGE_2_-induced hyperalgesia or rimonabant on capsaicin-induced pain [102]. The authors found that the methanol/methylene chloride extract administered orally at the doses of 250 and 500 mg/kg induced long lasting and significant antihyperalgesic effects on CFA-inflammatory and PSNL-induced neuropathic pain. The methanol/methylene chloride extract significantly reduced the mechanical hyperalgesia induced by PGE_2_ either when administered preventively or therapeutically [102]. The authors also found that the methanol/methylene chloride extract also significantly and time dependently inhibited the capsaicin-induced nociception. These studies show that* C. macrostachyus* extracts of the stem bark possess analgesic, anti-inflammatory, and antinociceptive properties corroborating the traditional use of the species in the treatment and management of different diseases and ailments, including pain and inflammation in tropical Africa.

### 6.9. Antileishmanial

Gelaw et al. [94] evaluated the antileishmanial activities of a compound, crotepoxide 3 isolated from chloroform extracts of* C. macrostachyus* against promastigotes and amastigotes form of* Leishmania aethiopica*. The result of the study revealed that observed IC_50_ values of crotepoxide 3 to be 219.7 and 229.70 *μ*g/ml against promastigotes and amastigotes, respectively, and therefore, less active when compared to the reference antileishmanial drugs amphotericin B and miltefosine with IC_50_ values of 0.03 and 0.12 *μ*g/ml, respectively [94].

### 6.10. Antioxidant

Teugwa et al. [86] evaluated antioxidant activities of methanolic leaf extracts of* C. macrostachyus* using 2,2-diphenyl-1-picrylhydrazyl (DPPH) scavenging methods. Methanolic leaf extract of* C. macrostachyus* showed antioxidant activity with IC_50_ value of 0.11 mg/ml. The documented antioxidant activities of* C. macrostachyus* leaf extracts are probably due to flavonoids and phenols that have been isolated from fruits, leaves, and roots [24, 30, 47, 51, 55, 84–89, 93]. Flavonoids and phenolic compounds found in plants are known to have antioxidant properties [110].

### 6.11. Antiplasmodial

Owuor et al. [103] evaluated antiplasmodial activities of dichloromethane leaf and stem extracts of* C. macrostachyus* using the SYBR Green I fluorescence assay (MSF assay) with mefloquine and chloroquine as positive controls. The dichloromethane leaf and stem extracts were active against chloroquine sensitive* Plasmodium falciparum* strain with IC_50_ value of 2.720 ± 0.627 *μ*g/ml [103]. Similarly, Bantie et al. [87] evaluated antiplasmodial activities of chloroform, methanol, and aqueous leaf extracts of* C. macrostachyus* using a rodent model of malaria. The rodent malaria parasite* Plasmodium berghei* was used to inoculate healthy male Swiss albino mice, 6–8 weeks old, and the parameters parasitemia, survival time, body weight, temperature, and packed cell volume were then determined using Peter's and Rane's tests [87]. Chemoprotective effect exerted by the extracts ranged between 12 and 91% and the chemotherapeutic effect of the extracts was in the range of 39–83%. The crude extracts prevented loss of weight and reduction in temperature but did not affect packed cell volume [87]. In another study, Mohammed et al. [104] evaluated antimalarial activities of the methanol and aqueous leaf extracts of* C. macrostachyus* using a 4-day suppressive standard test on* Plasmodium berghei*. Methanol and aqueous extracts of* C. macrostachyus* showed dose-dependent chemosuppressive effect at various doses in mice infected with* Plasmodium berghei* parasite while the crude methanol extracts of* C. macrostachyus* suppressed parasitaemia at all dose levels compared to the negative control groups but did not improve survival time [104]. The mice treated with the methanol and aqueous extracts at 600 mg/kg survived longer (10.60 ± 0.51 days for methanol extract and 9.60 ± 0.51 day for aqueous extract) than those in the negative control group with mean survival time of 6.2 ± 0.20 days [104]. The antimalarial activity test showed that* C. macrostachyus* exhibited significant antiplasmodial activity as evidenced by their ability to suppress* Plasmodium berghei* infection in mice in a dose-dependent manner, which may partly justify the claim by traditional practitioners about the use of these two plants against malaria. Mekonnen [89] also evaluated antiplasmodial activity of 80% methanol extract of the fruit and root of* C. macrostachyus* in a rodent model of malaria. The rodent malaria parasite* Plasmodium berghei* was used to inoculate healthy 8-week-old male Swiss albino mice and the parameters of parasitemia, survival time, body weight, temperature, and packed cell volume were determined using Peter's test and Rane's test [89]. Both extracts significantly inhibited parasitemia, increased survival time, prevented loss of weight and temperature, but did not affect the packed cell volume [89]. Results of this study suggest that the root and fruit extracts of* C. macrostachyus* have promising antiplasmodial activity against* Plasmodium berghei* in a dose-dependent manner, which supports the folkloric use of the plant for treating malaria.

### 6.12. Larvicidal

Karunamoorthi and Ilango [19] evaluated larvicidal activities of methanol leaf extracts of* C. macrostachyus* against late third instar larvae of* Anopheles arabiensis* Patton, a potent malaria vector. The larval mortality was observed 24 h of posttreatment. The methanol leaf extracts showed different degree of mortality against the malaria vector* Anopheles arabiensis* with LC_50_ and LC_90_ values of 89.25 and 224.98 ppm, respectively. These results establish that* C. macrostachyus* could serve as potent mosquito larvicidal agent against* Anopheles arabiensis* although its mode of actions and larvicidal efficiency under the field conditions should be investigated and determined.

### 6.13. Toxicity

The results of toxicity studies of aqueous and hydroalcoholic bark extracts of* C. macrostachyus* using albino mice showed LD_50_ value of 190.2 ± 15.7 mg/kg for aqueous extract and LD_50_ value of 87.5 ± 12.3 mg/kg for hydroalcoholic extract [73]. Desta [73] determined the median effective single dose of* C. macrostachyus* bark extract, that is, the dose that expelled* Taenia saginata* L. worms partially or totally in 50% of worm-infested human volunteers.* Croton macrostachyus* showed median effective single dose of 6.42 ± 0.82 and the number of hours that elapsed before partial or total expulsion of the worms following administration of* C. macrostachyus* was 12.9 ± 2.1 hours. Gadir et al. [106] evaluated oral toxicity of* C. macrostachyus* seeds in Nubian goat kids through clinical, hematological, and pathophysiological parameters. The Nubian goat kids were allotted as untreated controls and ground* C. macrostachyus* seeds were given to kids in repeated daily oral doses of 1 g/kg or 0.25 g/kg. Both oral dose levels of* C. macrostachyus* seeds were lethal for kids between days 7 and 21 and caused bloody diarrhoea, dyspnea, dehydration, loss in condition, paresis of the hind limbs, and recumbency before death [106]. Lesions in the affected animals included widespread hemorrhages and congestion, enterohepatonephrotoxicity, pulmonary hemorrhage, emphysema and cyanosis, tracheal froths, ascites, and hydropericardium. These lesions were accompanied by increases in the activity of serum AST, in the concentration of urea, and decreases in total protein and albumin, anemia, and leukopenia [106]. Moshi et al. [43] evaluated toxicity of root aqueous ethanol extracts of* C. macrostachyus* using the brine shrimp lethality test.* Croton macrostachyus* demonstrated moderate toxicity with concentration killing 50% (LC_50_) of the shrimps at 13.40 *μ*g/ml. These findings indicate the possibility that* C. macrostachyus* extracts may be toxic or contain useful cytotoxic compounds, which were not reported by the traditional healers. Wagate et al. [96] evaluated cytotoxicity of leaf and root methanolic extracts of* C. macrostachyus* using the brine shrimp lethality test.* Croton macrostachyus* showed LC_50_ value of 387 *μ*g/mL, which was considered to be relatively nontoxic [96].

Mbiantcha et al. [107] evaluated toxicity of aqueous and methylene chloride/methanol extracts of C.* macrostachyus* stem bark using* Artemia* spp. lethality assay, mice and Wistar rats. For the cytotoxicity study, the aqueous and organic extracts were administered to larvae of* Artemia* spp. and the number of deaths was determined after 6 hours and 24 hours, while for the acute study, the extracts were administered to mice. In the cytotoxicity study, aqueous and organic extracts showed LC_50_ values of 569 and 425 *μ*g/ml, respectively. In acute toxicity study, aqueous extract did not provoke death until the dose 16 g/kg, whereas the organic extract caused general behaviors, adverse effects, and mortality. Mortality increased with increasing doses, with LD_50_ values of 10.2 and 9.4 g/kg bwt, respectively, for male and female mice [107]. In another acute toxicity study in mice, the methanol leaf extract of* C. macrostachyus* at a single oral doses of 2 and 5 g/kg bwt caused no mortality within the first 24 h and up to 14 days observation period [87]. Results from the study suggested safety profile of this herbal extract in the study mice. Physical and behavioral observations of the experimental mice did not show any visible signs of overt toxicity such as lacrimation, loss of appetite, tremors, hair erection, salivation, and diarrhoea. These studies revealed that* C. macrostachyus* is not toxic in both acute and subacute tests at the tested doses of the extracts. In an* in vivo* study, the methanol extract of* C. macrostachyus* showed dose-dependent chemosuppressive effect at various dose levels, that is, 200 (21.1%), 400 (27.7%), and 600 (34.3%) mg/kg body weight in* Plasmodium berghei*-infected mice [104]. The mice treated with chloroquine were completely free from parasitemia on day 4 in all groups (100% suppression). The crude methanol extract of* C. macrostachyus* significantly suppressed parasitemia at all dose levels compared to the negative control groups (distilled water) but did not significantly prolong the survival time of infected mice. Similarly, the aqueous extract at the doses of 200, 400, and 600 mg/kg body weight significantly reduced % parasitemia (26.14, 30.50, and 50.53%, resp.) compared to the negative control group in this study. Mbunde et al. [27] evaluated toxicity of leaf dichloromethane extracts of* C. macrostachyus* using the brine shrimp lethality test.* Croton macrostachyus* demonstrated moderate toxicity with concentration killing 50% (LC_50_) of the shrimps at 12.94 *μ*g/ml [27]. Omosa et al. [108] also evaluated the cytotoxicity of dichloromethane and methanol (1 : 1) extract of* C. macrostachyus* stem bark using the resazurin reduction assay against CCRF-CEM leukemia cell line. The dichloromethane and methanol extract of* C. macrostachyus* stem bark displayed cytotoxicity towards leukemia CCRF-CEM cells with IC_50_ value of 60.6 *μ*g/mL [108]. Based on the cytotoxicity studies done on C. macrostachyus crude extracts [27, 43, 73, 96, 104, 106–108], it can be concluded that caution must be exercised in the use of the species as herbal medicine.

## 7. Conclusion

The present review summarizes the ethnomedicinal uses and recent findings on phytochemistry, pharmacology, and cytotoxicity of different extracts and compounds of* C. macrostachyus*. Alkaloids, amino acids, anthraquinones, carbohydrates, cardiac glycosides, coumarins, essential oil, fatty acids, flavonoids, phenolic compounds, phlobatannins, polyphenols, phytosteroides, saponins, sterols, tannins, terpenoids, unsaturated sterol, vitamin C, and withanoides have been demonstrated to be the main active ingredients of* C. macrostachyus*. Pharmacological studies have also focused on evaluating anthelmintic, antibacterial, antimycobacterial, antidiarrhoeal, antifungal, anticonvulsant and sedative, antidiabetic, anti-inflammatory, antileishmanial, antioxidant, antiplasmodial, larvicidal, and cytotoxicity activities of the different extracts and compounds isolated from* C. macrostachyus*. Future research should focus on the mechanisms of action of bioactive constituents of the species to illustrate the correlation between the ethnomedicinal uses and pharmacological properties of the species. Since previous studies have established that* C. macrostachyus* may contain potentially toxic compounds, there is need for detailed toxicological review of the crude extracts and pure compounds of the species. Lastly, since* C. macrostachyus* is widely used in combination with other plant species in various herbal concoctions, there is need for extensive research to evaluate synergistic effects of the different extracts or pure isolates to evaluate their ability to enhance the efficiency of the additive mixtures.

## Figures and Tables

**Table 1:** Ethnomedicinal uses of *Croton macrostachyus* in tropical Africa.

Use	Plant parts used	Country practiced	References
Abdominal pain	Leaf juice or decoction taken orally	Ethiopia, Tanzania	[26, 27]

Abdominal pain	Bark decoction mixed with roots of *Cucumis ficifolius* A. Rich. taken orally	Ethiopia	[28]

Abortifacient	Seeds eaten	Ethiopia	[29, 30]

Allergies	Pounded leaves rubbed on affected body parts	Ethiopia	[20, 31]

Amoebiasis	Leaf decoction taken orally	Ethiopia	[32]

Antidote for snake and scorpion venom	Bark, ground into powder and applied to affected body part	Ethiopia	[33]

Anthrax	Root decoction taken orally	Ethiopia	[34]

Ascariasis	Bark, leaf, or root decoction taken orally	Ethiopia	[35, 36]

Atopic eczema	Leaf decoction applied to affected body part	Ethiopia	[20]

Backache	Leaf and root decoction taken orally	Kenya	[37]

Bleeding or blood coagulant	Ashes, bark, and leaf juice applied on affected body part	Ethiopia, Kenya	[31, 32, 38, 39]

Bloat	Leaf juice taken orally	Ethiopia	[26]

Cancer	Leaf and root decoction taken orally	Ethiopia, Kenya	[37, 40]

Cleansing blood circulation system	Leaf decoction taken orally	Kenya	[41]

Cleansing digestive system	Leaf decoction taken orally	Kenya	[41]

Colds	Leaf decoction taken orally	Kenya	[41]

Constipation	Fruit, leaf, or root decoction taken orally	Ethiopia, Tanzania	[16, 30]

Cough	Leaf decoction taken orally	Kenya	[41]

Dandruff	Leaf fluid applied to the head	Ethiopia	[42]

Diabetes	Root decoction taken orally	Tanzania	[43]

Diarrhoea	Bark or leaf decoction taken orally	Ethiopia, Kenya	[44–47]

Diarrhoea	Leaf decoction mixed with *Trichilia* spp. and *Rhamnus prinoides* L'Hérit. taken orally	Ethiopia	[48]

Dry cough	Leaf or root decoction taken orally	Kenya	[37]

Dysentery	Leaf decoction mixed with *Trichilia* spp. and *Rhamnus prinoides* L'Hérit. taken orally	Ethiopia	[48]

Dysentery	Bark, leaf decoction taken orally	Ethiopia	[45, 47, 49]

Dysmenorrhoea	Bark or leaf decoction taken orally	Kenya	[46]

East coast fever	Leaf and root decoction taken orally	Kenya	[37]

Eczema	Leaf decoction applied to affected body part as an ointment	Ethiopia	[17]

Epilepsy	Leaf decoction taken orally	Cameroon, Ethiopia	[36, 50]

Fungal skin infection	Leaf ointment used on affected body parts	Ethiopia	[17, 18, 20, 28, 31, 45, 47, 51]

Gonorrhoea	Bark, fruit, leaf, or root decoction taken orally	Ethiopia	[31, 34–36, 52, 53]

Gum ailment	Leaf decoction rubbed on gums	Ethiopia	[34]

Headache	Root decoction rubbed on affected part	Uganda	[54]

Hemorrhage	Leaf sap rubbed on affected part	Ethiopia	[34]

Hepatitis	Root decoction taken orally	Ethiopia	[36]

Insomnia	Leaf decoction taken orally	Cameroon	[50]

Jaundice	Leaf decoction taken orally	Ethiopia	[28]

Leprosy	Sap applied on affected body part	Ethiopia	[55, 56]

Malaria	Bark, leaf, or root decoction taken orally or smoke from burnt leaves inhaled	Ethiopia, Kenya	[26, 28, 37, 44, 55–59]

Malaria	Leaf decoction mixed with *Allium sativum* L. bulb roasted with butter, allowed to brew overnight and taken orally	Ethiopia	[60, 61]

Measles	Fruit, leaf, or root decoction taken orally	Ethiopia	[55, 62]

Mosquito repellent	Smoke from burnt bark or leaves repel mosquitos	Ethiopia	[38]

Obesity	Leaf and root decoction taken orally	Kenya	[37]

Pneumonia	Bark, leaf, or root decoction taken orally	Cameroon, Kenya	[37, 63]

Protection against witchcraft	Whole plant	Uganda	[64]

Purgative	Fruit, leaf, root, or seed decoction taken orally	Cameroon, Kenya, Somalia, Tanzania	[16, 37, 65, 66]

Retained placenta	Leaf infusion taken orally	Ethiopia	[56]

Rheumatism	Leaf decoction taken orally	Ethiopia	[31]

Ringworm	Bark, leaf decoction rubbed on affected body part	Ethiopia, Kenya, Tanzania	[27, 31, 38, 46, 60, 61, 67, 68]

Skin cancer	Leaf decoction applied on affected part	Ethiopia	[67]

Skin diseases	Leaf and root decoction applied on affected body parts	Ethiopia, Kenya, Tanzania	[27, 37, 55]

Skin diseases	Fruit decoction mixed with *Hagenia abyssinica* (Bruce) J. F. Gmel. applied on affected body parts	Ethiopia	[69]

Skin rash	Mixed with egg yolk and applied to the skin	Ethiopia	[42]

Snake bite	Root decoction taken orally	Ethiopia	[70]

Sorcery	Leaf and root decoction	Kenya	[37]

Stomach ache	Bark, leaf, or root decoction taken orally	Ethiopia, Uganda	[34, 45, 49, 67, 71]

Struck by lightning	Patient bathed with leaf, fruit, and root decoction	Uganda	[54]

Tape worms	Bark decoction mixed with stems of *Glinus lotoides* L. taken orally	Ethiopia	[17]

Tape worms	Leaf decoction mixed with *Baccharoides calvoana* subsp. *meridonalis* (Wild) Isawumi, El-Ghazaly & B. Nord, *Vernonia auriculifera* Hiern, and *Salvia nilotica* Juss. ex Jacq. taken orally	Rwanda	[72]

Tape worms	Bark decoction mixed with *Morella salicifolia* subsp. *mildbraedii* (Engl.) Verdc. & Polhill, *Olea capensis *subsp. *macrocarpa* (C. H. Wright) Verdc., and *Elaeis guineensis* Jacq. taken orally	Rwanda	[72]

Tape worms	Bark, fruit, leaf, root, or seeds decoction taken orally	Ethiopia, Tanzania, Uganda	[16, 29, 34, 35, 52, 55, 71, 73]

Typhoid	Bark, leaf, or root decoction taken orally	Cameroon, Kenya	[37, 62, 74]

Ulcers	Bark powder dressing	Ethiopia	[17]

Venereal diseases	Fruits and root decoction taken orally	Ethiopia	[29, 30]

Warts	Leaf and twig sap applied on affected body parts	Kenya	[68]

Weakness in pregnancy	Patient tie the waist with a tree branch	Uganda	[54]

Wounds	Bark, leaf, and twig sap applied directly on wounds	Ethiopia, Kenya, Tanzania	[20, 26, 27, 32, 45, 47, 55, 67, 68]
	Leaf sap mixed with coconut (*Cocos nucifera* L.) milk and applied on wounds	Ethiopia	[44]
	Leaf decoction mixed with *Cynoglossum lanceolatum* Forssk. and *Dodonaea viscosa* subsp. *angustifolia* (L. f.) J. G. West applied on wounds	Ethiopia	[17]

*Ethnoveterinary medicine*			

Abdominal pain	Bark decoction	Ethiopia	[75, 76]

Blackleg	Leaf decoction	Ethiopia	[44, 77]

Bleeding	Leaf decoction	Ethiopia	[21]

Bloat	Bark, leaf or twig decoction	Ethiopia	[48, 75, 76, 78]

Colic	Bark decoction	Ethiopia	[75, 76]

Constipation	Leaf decoction	Ethiopia	[77]

Dermatophilosis	Leaf decoction	Ethiopia	[21]

Epilepsy in cattle	Leaf decoction	Ethiopia	[36]

External parasites	Leaf decoction mixed with *Trichilia* spp. and *Rhamnus prinoides* L'Hérit. applied topically	Ethiopia	[48]

Fever in cows	Bark, leaf decoction	Kenya	[46]

Mange	Leaf decoction	Ethiopia	[21]

Rabies	Bark decoction mixed with leaves of *Juniperus procera* Endl., stems of *Eragrostis tef *(Zucc.) Trotter, and roots of *Cyphostemma cyphopetalum* (Fresen.) Desc. ex Wild & Drummond, *Solanum anguivi* Lam., and *Solanum marginatum* L.	Ethiopia	[17]

Rabies	Root and stem bark, chewing and swallowing the juice three times a day	Ethiopia	[23, 35, 38, 48]

Rectum prolapse	Root decoction	Ethiopia	[48]

Ringworm	Leaf decoction applied directly	Ethiopia, Kenya	[21, 68, 78]

Scabies	Leaf decoction	Ethiopia	[21]

Skin diseases (dermatophilosis)	Root decoction	Ethiopia	[48]

Streptothricosis	Leaves pounded and applied on skin surface	Ethiopia	[26]

Swelling of nose in mule	Leaf decoction	Ethiopia	[36]

Tick prevention and control	Bark, leaf, or root decoction used	Kenya	[79]

Warts	Sap applied directly	Kenya	[68]

Wound	Leaf decoction applied directly	Ethiopia, Kenya	[21, 68]

*Other uses*			

Beehives		Kenya, Tanzania	[16, 57]

Firewood		Ethiopia, Tanzania, Uganda	[16, 40, 80]

Fodder		Uganda	[80]

Pesticide	Leaves mixed with *Nicotiana tabacuum *L. used as pesticide for maize stalk borers and aphids	Kenya	[57]

Shade		Uganda	[80]

Source of nectar		Kenya	[81]

Water pots, wood carving, tool handles, timber, stools		Cameroon, Ethiopia, Tanzania	[16, 40, 63, 65]

**Table 2:** Chemical compounds isolated and characterized from *Croton macrostachyus*.

Number	Compound	Plant part(s)	Reference(s)
	**Triterpene**		
**1**	Betulin	Stem bark, twigs	[83, 92, 93]
**2**	Lupeol	Stem bark, twigs	[83, 92, 93]
**6**	3*β*-Acetoxy taraxer-14-en-28-oic acid	Roots	[90]
**16**	Lupenone	Twigs	[93]
**17**	Betulinic acid	Twigs	[93]
**18**	28-O-Acetylbetulin	Twigs	[93]
**19**	Lupeol acetate	Twigs	[93]
**20**	Zeorin	Twigs	[93]
	**Cyclohexane diepoxide**		
**3**	Crotepoxide	Fruits, stem bark, twigs	[83, 91, 94]
	**Phytosterol**		
**4**	*β*-Sitosterol	Stem bark, twigs	[83, 93]
**5**	Stigmasterol	Stem bark, twigs	[83, 93]
**25**	*β*-Sitosterol palmitate	Twigs	[93]
	**Diterpenoids**		
**7**	Trachyloban-19-oic acid	Roots	[90]
**8**	Trachyloban-18-oic acid	Roots	[90]
**9**	Neoclerodan-5,10-en-19,6*β*;20,12-diolide	Roots	[90]
**10**	3*α*,19-Dihydroxytrachylobane	Roots	[90]
**11**	3*α*,18,19-Trihydroxytrachylobane	Roots	[90]
**12**	Crotomacrine	Fruits	[91]
**13**	Floridolide A	Stem bark	[92]
**14**	Hardwickic acid	Stem bark	[92]
**15**	12-oxo-Hardwickic acid	Stem bark	[92]
	**Phenolic compounds**		
**21**	Benzoic acid	Twigs	[93]
**22**	Methyl gallate	Twigs	[93]
**23**	Methyl 2,4-dihydroxy-3,6-dimethylbenzoate	Twigs	[93]
**24**	Lichexanthone	Twigs	[93]

## References

[1] Smith A. R., Polhill R. M. (1987). Euphorbaceae. *Flora of Tropical East Africa*.

[2] Gilbert M. G., Edwards S., Mesfin T., Hedberg I. (1995). Flora of Ethiopia and Eritrea. *Euphorbiaceae*.

[3] Wakjira K., Negash L. (2013). Germination responses of Croton macrostachyus (Euphorbiaceae) to various physico-chemical pretreatment conditions. *South African Journal of Botany*.

[4] Bekele-Tesemma A., Birnie A., Tengnäs B. (1993). Useful trees and shrubs for ethiopia: identification, propagation and management for agricultural and pastoral communities. *Technical Handbook no. 5, Regional Soil Conservation Unit/SIDA*.

[5] Hines D. A., Eckman K. (1993). *Indigenous Multipurpose Trees for Tanzania: Uses and Economic Benefits to the People*.

[6] Maundu P., Tengnäs B.

[7] Dechasa J. (1999). Influence of Croton macrostachyus on maize yield: Traditional inter-crop farming system. *Walia*.

[8] Oliver-Bever B. (1986). *Medicinal plants in tropical West Africa*.

[9] Burkill H. M. (1994). *The Useful Plants of West Tropical Africa*.

[10] Neuwinger H. D. (2000). *African Traditional Medicine: A Dictionary of Plant Use and Applications*.

[11] Mairura F. S., Schmelzer G. H., Gurib-Fakim A. (2008). *Croton macrostachyus* Hochst. ex Delile. *Plant Resources of Tropical Africa: Medicinal Plants 1*.

[12] WHO Traditional Medicine Strategy http://www.who.int/medicines/publications/traditionalpolicy/en/index.html.

[13] Maroyi A. (2014). Alternative medicines for HIV/AIDS in resource-poor settings: Insight from traditional medicines use in sub-Saharan Africa. *Tropical Journal of Pharmaceutical Research*.

[14] Richardson A., King K. (2010). *Plants of Deep South Texas: A Field Guide to the Woody and Flowering Species*.

[15] Orwa C., Mutua A., Kindt R., Jamnadass R., Anthony S. http://www.worldagroforestry.org/sites/treedbs/treedatabases.asp.

[16] Lovett J. C., Ruffo C. K., Gereau R. E. (2006). *Field Guide to the Moist Forest Trees of Tanzania*.

[17] Teklehaymanot T., Giday M., Medhin G., Mekonnen Y. (2007). Knowledge and use of medicinal plants by people around Debre Libanos monastery in Ethiopia. *Journal of Ethnopharmacology*.

[18] Teklehaymanot T. (2009). Ethnobotanical study of knowledge and medicinal plants use by the people in Dek Island in Ethiopia. *Journal of Ethnopharmacology*.

[19] Karunamoorthi K., Ilango K. (2010). Larvicidal activity of Cymbopogon citratus (DC) Stapf. and Croton macrostachyus Del. against Anopheles arabiensis Patton, a potent malaria vector. *European Review for Medical and Pharmacological Sciences*.

[20] Lulekal E., Asfaw Z., Kelbessa E., Van Damme P. (2013). Ethnomedicinal study of plants used for human ailments in Ankober District, North Shewa Zone, Amhara Region, Ethiopia. *Journal of Ethnobiology and Ethnomedicine*.

[21] Lulekal E., Asfaw Z., Kelbessa E., Van Damme P. (2014). Ethnoveterinary plants of Ankober District, North Shewa Zone, Amhara Region, Ethiopia. *Journal of Ethnobiology and Ethnomedicine*.

[22] Dubale A. A., Chandravanshi B. S., Gebremariam K. F. (2015). Levels of major and trace metals in the leaves and infusions of Croton macrostachyus. *Bulletin of the Chemical Society of Ethiopia*.

[23] Pagadala V. K., Tsegaye B., Kebede N., Elias T., Gemachu G. (2015). Significance of traditional medicinal plants used for treatment of rabies at Ambo town. *Medicinal and Aromatic Plants*.

[24] Degu A., Engidawork E., Shibeshi W. (2016). Evaluation of the anti-diarrheal activity of the leaf extract of Croton macrostachyus Hocsht. ex Del. (Euphorbiaceae) in mice model. *BMC Complementary and Alternative Medicine*.

[25] Friis I. (1992). *Forests and Forest Trees of Northeast Tropical Africa: Their Natural Habitats and Distribution Patterns in Ethiopia, Djibouti and Somalia*.

[26] Tadesse M., Hunde D., Getachew Y. (2005). Survey of medicinal plants used to treat human diseases in Seka Chekorsa, Jimma zone, Ethiopia. *Ethiopian Journal of Health Sciences*.

[27] Mbunde M. V. N., Innocent E., Mabiki F., Andersson P. G. (2017). Ethnobotanical survey and toxicity evaluation of medicinal plants used for fungal remedy in the southern highlands of Tanzania. *Journal of Intercultural Ethnopharmacology*.

[28] Teklay A., Abera B., Giday M. (2013). An ethnobotanical study of medicinal plants used in Kilte Awulaelo district, Tigray Region of Ethiopia. *Journal of Ethnobiology and Ethnomedicine*.

[29] Wilson R. T., Mariam W. G. (1979). Medicine and magic in central tigre: A contribution to the ethnobotany of the ethiopian plateau. *Economic Botany*.

[30] Eguale T., Tilahun G., Gidey M., Mekonnen Y. (2006). In vitro anthelmintic activities of four Ethiopian medicinal plants against Haemonchuscontortus , Pharmacologyonline. *Pharmacologyonline*.

[31] Yineger H., Yewhalaw D., Teketay D. (2008). Ethnomedicinal plant knowledge and practice of the Oromo ethnic group in southwestern Ethiopia. *Journal of Ethnobiology and Ethnomedicine*.

[32] Parvez N., Yadav S. (2010). Ethnopharmacology of single herbal preparations of medicinal plants in Asendabo district, Jimma, Ethiopia. *Indian Journal of Traditional Knowledge*.

[33] Flatie T., Gedif T., Asres K., Gebre-Mariam T. (2009). Ethnomedical survey of Berta ethnic group assosa zone, benishangul-gumuz regional state, mid-west Ethiopia. *Journal of Ethnobiology and Ethnomedicine*.

[34] Wondimu T., Asfaw Z., Kelbessa E. (2007). Ethnobotanical study of medicinal plants around 'Dheeraa' town, Arsi Zone, Ethiopia. *Journal of Ethnopharmacology*.

[35] Giday M., Teklehaymanot T., Animut A., Mekonnen Y. (2007). Medicinal plants of the Shinasha, Agew-awi and Amhara peoples in northwest Ethiopia. *Journal of Ethnopharmacology*.

[36] Lulekal E., Kelbessa E., Bekele T., Yineger H. (2008). An ethnobotanical study of medicinal plants in Mana Angetu District, southeastern Ethiopia. *Journal of Ethnobiology and Ethnomedicine*.

[37] Jeruto P., Mutai C., Ouma G., Lukhoba C. (2011). An inventory of medicinal plants that the people of Nandi use to treat malaria. *Journal of Animal and Plant Science*.

[38] Megersa M., Asfaw Z., Kelbessa E., Beyene A., Woldeab B. (2013). An ethnobotanical study of medicinal plants in Wayu Tuka District, East Welega Zone of Oromia Regional State, West Ethiopia. *Journal of Ethnobiology and Ethnomedicine*.

[39] Karemu P. G., Kenji G. M., Gachanja A. N., Keriko J. M., Mungai G. (2007). Traditional medicines among the Embu and Mbeere peoples of Kenya. *African Journal of Traditional, Complementary and Alternative Medicines*.

[40] Reta R. (2016). Useful plant species diversity in homegardens and its contribution to household food security in Hawassa city, Ethiopia. *African Journal of Plant Science*.

[41] Adongo O. S. (2013). *Medicinal plants of Chuka community in Tharaka Nithi county, Kenya and some of their selected essential elements [MSc dissertation] [dissertation, thesis]*.

[42] d'Avigdor E., Wohlmuth H., Asfaw Z., Awas T. (2014). The current status of knowledge of herbal medicine and medicinal plants in Fiche, Ethiopia. *Journal of Ethnobiology and Ethnomedicine*.

[43] Moshi M. J., Cosam J. C., Mbwambo Z. H., Kapingu M., Nkunya M. H. H. (2004). Testing beyond ethnomedical claims: brine shrimp lethality of some tanzanian plants. *Pharmaceutical Biology*.

[44] Mesfin F., Seta T., Assefa A. (2014). An ethnobotanical study of medicinal plants in Amaro Woreda, Ethiopia. *Ethnobotany Research and Applications *.

[45] Amuamuta A., Mekonnen Z., Gebeyehu E. (2015). Traditional therapeutic uses and phytochemical screening of some selected indigenous medicinal plants from Northwest Ethiopia. *African Journal of Pharmacology and Therapeutics*.

[46] Okello S. V., Nyunja R. O., Netondo G. W., Onyango J. C. (2010). Ethnobotanical study of medicinal plants used by sabaots of mt. Elgon kenya. *African Journal of Traditional, Complementary and Alternative Medicines*.

[47] Amuamuta A., Mekonnen Z., Gebeyehu E. (2014). Therapeutic usage and phytochemical screening study on some selected indigenous medicinal plants from Zegie and Lake Tana areas, Northwest Ethiopia. *European Journal of Applied Sciences*.

[48] Eshetu G. R., Dejene T. A., Telila L. B., Bekele D. F. (2015). Ethnoveterinary medicinal plants: preparation and application methods by traditional healers in selected districts of southern Ethiopia. *Veterinary World*.

[49] Temam T., Dillo A. (2016). Ethnobotanical study of medicinal plants of Mirab-Badwacho district, Ethiopia. *Journal of BioSciences and Biotechnology*.

[50] Bum E. N., Ngah E., Mune R. M. N. (2012). Decoctions of Bridelia micrantha and Croton macrostachyus may have anticonvulsant and sedative effects. *Epilepsy Behavior*.

[51] Busse H., Tefera G. (2013). *Handbook of Sidama Traditional Medicinal Plants*.

[52] Kidane B., van Andel T., van der Maesen L. J. G., Asfaw Z. (2014). Use and management of traditional medicinal plants by Maale and Ari ethnic communities in southern Ethiopia. *Journal of Ethnobiology and Ethnomedicine*.

[53] Mesfin T., Aberra G., Asfaw D. (2012). In vitro anti-Neisseria gonorrhoeae activity of Albizia gummifera and Croton macrostachyus. *Pharmacologyonline*.

[54] Tugume P., Kakudidi E. K., Buyinza M. (2016). Ethnobotanical survey of medicinal plant species used by communities around Mabira Central Forest Reserve, Uganda. *Journal of Ethnobiology and Ethnomedicine*.

[55] Geyid A., Abebe D., Debella A. (2005). Screening of some medicinal plants of Ethiopia for their anti-microbial properties and chemical profiles. *Journal of Ethnopharmacology*.

[56] Suleman S., Alemu T. (2012). A survey on utilization of ethnomedicinal plants in Nekemte town, East Wellega (Oromia), Ethiopia. *Journal of Herbs, Spices & Medicinal Plants*.

[57] Mwamidi D. M., Mwasi S. M., Nunow A. A. (2012). Indigenous knowledge of Taita community in the use and conservation of medicinal plants: The case of Taita hills, Kenya. *Journal Bio Innovation*.

[58] Jeruto P., Mutai C., Ouma G., Lukhoba C., Nyamaka R. L., Manani S. D. (2010). Ethnobotanical survey and propagation of some endangered medicinal plants from south Nandi district of Kenya. *Journal of Animal and Plant Science*.

[59] Mukungu N., Abuga K., Okalebo F., Ingwela R., Mwangi J. (2016). Medicinal plants used for management of malaria among the Luhya community of Kakamega East sub-County, Kenya. *Journal of Ethnopharmacology*.

[60] Mesfin F., Demissew S., Teklehaymanot T. (2009). An ethnobotanical study of medicinal plants in Wonago Woreda, SNNPR, Ethiopia. *Journal of Ethnobiology and Ethnomedicine*.

[61] Bekele G., Reddy P. R. (2015). Ethnobotanical study of medicinal plants used to treat human ailments by Guji Oromo tribes in Abaya district, Borana, Oromia, Ethiopia. *Universal Journal of Plant Science*.

[62] Obey J. K., von Wright A., Orjala J., Kauhanen J., Tikkanen-Kaukanen C. (2016). Antimicrobial activity of *Croton macrostachyus* stem bark extracts against several human pathogenic bacteria. *Journal of Pathogens*.

[63] Focho D. A., Newu M. C., Anjah M. G., Nwana F. A., Ambo F. B. (2009). Ethnobotanical survey of trees in Fundong, Northwest Region, Cameroon. *Journal of Ethnobiology and Ethnomedicine*.

[64] Kakudidi E. K. (2004). Cultural and social uses of plants from and around Kibale National Park, Western Uganda. *African Journal of Ecology, Supplement*.

[65] Simbo D. J. (2010). An ethnobotanical survey of medicinal plants in Babungo, Northwest Region, Cameroon. *Journal of Ethnobiology and Ethnomedicine*.

[66] Mazzanti G., Bolle P., Martinoli L. (1987). Croton macrostachys, a plant used in traditional medicine: purgative and inflammatory activity. *Journal of Ethnopharmacology*.

[67] Regassa R. (2013). Diversity and conservation status of some economically valued indigenous medicinal plants in hawassa college of teacher education campus, Southern Ethiopia. *International Journal of Advanced Research*.

[68] Njoroge G. N., Bussmann R. W. (2007). Ethnotherapeautic management of skin diseases among the Kikuyus of Central Kenya. *Journal of Ethnopharmacology*.

[69] Zerabruk S., Yirga G. (2012). Traditional knowledge of medicinal plants in Gindeberet district, Western Ethiopia. *South African Journal of Botany*.

[70] Giday M., Asfaw Z., Woldu Z. (2009). Medicinal plants of the meinit ethnic group of ethiopia: an ethnobotanical study. *Journal of Ethnopharmacology*.

[71] Hamill F. A., Apio S., Mubiru N. K. (2000). Traditional herbal drugs of southern Uganda, I. *Journal of Ethnopharmacology*.

[72] Nahayo A., Bigendako M. J., Fawcett K., Gu Y. (2010). Ethnobotanic study around Volcanoes National Park, Rwanda. *New York Science Journal*.

[73] Desta B. (1995). Ethiopian traditional herbal drugs. Part I: studies on the toxicity and therapeutic activity of local taenicidal medications. *Journal of Ethnopharmacology*.

[74] Tsobou R., Mapongmetsem P. M., Van Damme P. (2013). Medicinal plants used against typhoid fever in Bamboutos division, western Cameroon. *Ethnobotany Research and Applications *.

[75] Tekle Y. (2014). An ethno-veterinary botanical survey of medicinal plants in Kochore district of Gedeo Zone, Southern Nations Nationalities and Peoples Regional State (SNNPRs), Ethiopia. *Journal of Science and Innovative Research*.

[76] Tekle Y. (2015). Medicinal plants in the ethno veterinary practices of bensa woreda, Southern Ethiopia. *Open Access Library Journal*.

[77] Kidane B., Van Der Maesen L. J. G., Van Andel T., Asfaw Z. (2014). Ethnoveterinary medicinal plants used by the Maale and Ari ethnic communities in southern Ethiopia. *Journal of Ethnopharmacology*.

[78] Sori T., Bekana M., Adugna G., Kelbessa E. (2004). Medicinal plants in the ethnoveterinary practices of Borana pastoralists, Southern Ethiopia. *International Journal of Applied Research in Veterinary Medicine*.

[79] Wanzala W., Takken W., Mukabana W. R., Pala A. O., Hassanali A. (2012). Ethnoknowledge of Bukusu community on livestock tick prevention and control in Bungoma district, western Kenya. *Journal of Ethnopharmacology*.

[80] Sebukyu V. B., Mosango D. M. (2012). Adoption of agroforestry systems by farmers in Masaka District of Uganda. *Ethnobotany Research and Applications *.

[81] Ichikawa M. (1987). A preliminary report on the ethnobotany of the Suiei Dorobo in northen Kenya. *African Study Monographs*.

[82] Agisho H., Osie M., Lambore T. (2014). Traditional medicinal plants utilization, management and threats in Hadiya Zone, Ethiopia. *Journal of Medinal Plant Studies*.

[83] Addae-Mensah I., Muriuki G., Karanja G., Wandera C., Waibel R., Achenbach H. (1992). Constituents of the stem bark and twigs of *Croton macrostachyus*. *Fitoterapia*.

[84] Kamanyi A., Mbiantcha M., Nguelefack T. B. (2009). Anti-nociceptive and anti-inflammatory activities of extracts from the stem bark of Croton macrostachyus (Euphorbiaceae) in mice and rats. *Journal of Complementary and Integrative Medicine*.

[85] Mesfin T., Aberra G., Asfaw D. (2010). In vitro anti-Neisseria gonorrhoeae activity of *Albizia gummifera* and *Croton macrostachyus*. *Revista Cenic Ciencias Biológicas*.

[86] Teugwa M. C., Sonfack D. C., Fokom R., Penlap B. V., Amvam Z. P. H. (2013). Antifungal and antioxidant activity of crude extracts of three medicinal plants from Cameroon pharmacopeia. *Journal of Medicinal Plant Research*.

[87] Bantie L., Assefa S., Teklehaimanot T., Engidawork E. (2014). In vivo antimalarial activity of the crude leaf extract and solvent fractions of *Croton macrostachyus* Hocsht. (Euphorbiaceae) against *Plasmodium berghei* in mice. *BMC Complementary and Alternative Medicine*.

[88] Arika W. M., Abdirahman Y. A., Mawia M. A. (2015). In vivo antidiabetic activity of the aqueous leaf extract of *Croton macrostachyus* in alloxan induced diabetic mice. *Pharmaceutica Analytica Acta*.

[89] Mekonnen L. B. (2015). In vivo antimalarial activity of the crude root and fruit extracts of Croton macrostachyus (Euphorbiaceae) against Plasmodium berghei in mice. *Journal of Traditional and Complementary Medicine*.

[90] Kapingu M. C., Guillaume D., Mbwambo Z. H., Moshi M. J., Uliso F. C., Mahunnah R. L. A. (2000). Diterpenoids from the roots of *Croton macrostachys*. *Phytochemistry*.

[91] Tane P., Tatsimo S., Connolly J. D. (2004). Crotomacrine, a new clerodane diterpene from the fruits of *Croton macrostachyus*. *Tetrahedron Letters*.

[92] Tene M., Ndontsa B. L., Tane P., Tamokou J. D. (2009). Antimicrobial diterpenoids and triterpenoids from the stem bark of *Croton macrostachys*. *International Journal of Biological and Chemical Sciences*.

[93] Tala M. F., Tan N.-H., Ndontsa B. L., Tane P. (2013). Triterpenoids and phenolic compounds from Croton macrostachyus. *Biochemical Systematics and Ecology*.

[94] Gelaw H., Adane L., Tariku Y., Hailu A. (2012). Isolation of crotepoxide from berries of *Croton macrostachyus* and evaluation of its anti-lishmanial activity. *Journal of Pharmacognosy and Phytochemistry*.

[95] Aleme H., Awetahegne Y., Tesfaye A. (2015). In vitro antihelmintic activities of four medicinal plants against Haemonchuscontortus. *Scientific Research Journal*.

[96] Wagate G. C., Gakuya W. D., Nanyingi M. O., Njonge F. K., Mbaria J. M. (2008). Antibacterial and cytotoxic activity of Kenyan medicinal plants. *Memórias doInstituto Oswaldo Cruz Rio de Janeiro*.

[97] Wagate C. G., Mbaria J. M., Gakuya D. W. (2010). Screening of some Kenyan medicinal plants for antibacterial activity. *Phytotherapy Research*.

[98] Belay G., Tariku Y., Kebede T., Hymete A., Mekonnen Y. (2011). Ethnopharmacological investigations of essential oils isolated from five Ethiopian medicinal plants against eleven pathogenic bacterial strains. *Phytopharmacology*.

[99] Taye B., Giday M., Animut A., Seid J. (2011). Antibacterial activities of selected medicinal plants in traditional treatment of human wounds in Ethiopia. *Asian Pacific Journal of Tropical Biomedicine*.

[100] Sendeku W., Alefew B., Mengiste D. (2015). Antibacterial activity of *Croton macrostachyus* against some selected pathogenic bacteria. *Biotechnoloy International*.

[101] Abera A., Lemessa F., Muleta D. (2011). The antifungal activity of some medicinal plants against coffee berry disease caused by colletotrichum kahawae. *International Journal of Agricultural Research*.

[102] Nguelefack T. B., Dutra R. C., Paszcuk A. F., de Andrade E. L., Calixto J. B. (2015). TRPV1 channel inhibition contributes to the antinociceptive effects of Croton macrostachyus extract in mice. *BMC Complementary and Alternative Medicine*.

[103] Owuor B. O., Ochanda J. O., Kokwaro J. O. (2012). In vitro antiplasmodial activity of selected Luo and Kuria medicinal plants. *Journal of Ethnopharmacology*.

[104] Mohammed T., Erko B., Giday M. (2014). Evaluation of antimalarial activity of leaves of Acokanthera schimperi and Croton macrostachyus against Plasmodium berghei in Swiss albino mice.. *BMC Complementary and Alternative Medicine*.

[105] Gemechu A., Giday M., Worku A., Ameni G. (2013). In vitro anti-mycobacterial activity of selected medicinal plants against *Mycobacterium tuberculosis* and *Mycobacterium bovis* strains. *BMC Complementary and Alternative Medicine*.

[106] Gadir W. S. A., Onsa T. O., Ali W. E. M., El Badwi S. M. A., Adam S. E. I. (2003). Comparative toxicity of *Croton macrostachys, Jatropha curcas and Piper abyssinica* seeds in Nubian goats. *Small Ruminant Research*.

[107] Mbiantcha M., Nguelefack T. B., Ndontsa B. L., Tane P., Kamanyi A. Preliminary assessment of toxicity of *Croton macrostachyus* stem bark (Euphorbiaceae) extracts. *International Journal of Pharmaceutical, Chemistry and Biological Sciences*.

[108] Omosa L. K., Midiwo J. O., Masila V. M. (2016). Cytotoxicity of 91 Kenyan indigenous medicinal plants towards human CCRF-CEM leukemia cells. *Journal of Ethnopharmacology*.

[109] Semenya S. S., Maroyi A. (2013). Medicinal plants used for the treatment of tuberculosis by Bapedi traditional healers in the Limpopo Province, South Africa. *African Journal of Traditional, Complementary and Alternative Medicine*.

[110] Miguel M. G., Nunes S., Dandlen S. A., Cavaco A. M., Antunes M. D. (2014). Phenols, flavonoids and antioxidant activity of aqueous and methanolic extracts of propolis (*Apis mellifera* L.) from Algarve, South Portugal. *Food Science and Technology*.

